# The Association between Plant-Based Diets and Dietary Patterns with Cardiometabolic Risk in a Sample of Commercial Taxi Drivers in South Africa

**DOI:** 10.3390/nu15071789

**Published:** 2023-04-06

**Authors:** Tatum Lopes, Annalise Edith Zemlin, Machoene Derrick Sekgala, Zandile June-Rose Mchiza, Rajiv Timothy Erasmus, Andre Pascal Kengne

**Affiliations:** 1 Non-Communicable Diseases Research Unit, South African Medical Research Council, Cape Town 7505, South Africa; 2 Division of Chemical Pathology, Department of Pathology, Faculty of Medicine and Health Sciences, University of Stellenbosch, Cape Town 7505, South Africa; 3 National Health Laboratory Service (NHLS), Tygerberg Hospital, Cape Town 7505, South Africa; 4 School of Public Health, University of the Western Cape, Bellville 7535, South Africa; 5 Human and Social Capabilities, Human Sciences Research Council, Cape Town 8000, South Africa; 6 Department of Medicine, University of Cape Town, Cape Town 7925, South Africa

**Keywords:** taxi drivers, plant-based diets, dietary patterns, cardiometabolic risk, South Africa

## Abstract

The consumption of unhealthy foods and a sedentary lifestyle predispose individuals to non-communicable diseases. This study investigated the distribution and the association of plant-based diets (PBDs) and dietary patterns in relation to the cardiometabolic risks in commercial taxi drivers. A cross-sectional analysis was conducted among males (≥19 years) who consumed street foods sold by vendors in the Cape Metropole. A validated questionnaire was administered, including a quantified 24 h dietary recall, and fasting blood samples were collected for biochemical analyses. Statistical analyses were performed to investigate the association between dietary habits and cardiometabolic risks, while adjusting for confounding variables. The analytic sample consisted of 189 males with a median age of 38 years. The taxi drivers who ranked in the top-third of the healthy plant-based diet index (hPDI) had a 1–4% lower likelihood of having raised triglycerides (TG). Furthermore, consumption patterns including refined grains and meat conferred a 33% lower likelihood of dysglycaemia (*p* = 0.049), while fish/seafood, potatoes, and vegetables conferred a 43% greater likelihood of low high-density lipoprotein cholesterol (HDL-C) (*p* = 0.026) and 44% greater probability of raised low-density lipoprotein cholesterol (LDL-C) (*p* = 0.027). Consumption patterns, including sugar-sweetened beverages and eggs, conferred a 37% greater probability of hypertension (*p* = 0.047) and 53% greater likelihood of subclinical inflammation (*p* = 0.017). These preliminary findings require larger and more elaborate studies to explore the associations between PBDs and dietary patterns in at-risk African populations, with or without sedentary lifestyles, and exposure to unhealthy food environments.

## 1. Introduction

Commercial taxi drivers form an integral part of the South African community, since they contribute to the economy by offering daily transport services to many commuters in urban settings [1]. Due to the growing population and urbanisation, the demand for public transportation has increased, yet it differs between high-income countries (HICs) and low-to-middle income countries (LMICs) [2,3,4]. This demand plays a role in shaping the working environment of commercial taxi drivers, often resulting in long working hours, which promote fatigue and may have adverse effects on their well-being.

In addition to the stressful working conditions of commercial taxi drivers, other lifestyle factors, such as sedentary behaviour and unhealthy food consumption including processed foods that are high in fat and sugar, make this population susceptible to health problems, such as non-communicable diseases (NCDs) [1,5]. Nutrition-related NCDs include cardiometabolic risk factors, such as hypertension, dysglycaemia, dyslipidaemia, and obesity, which have become more prevalent among taxi drivers [5,6,7]. The literature comparing this issue in HICs and LMICs has shown that the development of cardiometabolic diseases was significantly influenced by sociodemographic determinants, such as age and level of education, amongst others [8,9,10].

Other factors, such as urbanisation and modernised food environments, have further promoted the development of cardiometabolic risk factors worldwide [11]. Many LMICs have urban food environments that supply street foods, which are usually sold by vendors and easily accessible to consumers, such as commercial taxi drivers [12,13,14]. Street foods consist of various items ranging from relatively healthy (e.g., home-cooked meals) to unhealthy plant and animal foods that are highly processed (e.g., with high salt, fat, and sugar contents) [14]. The dietary habits of commercial taxi drivers operating in urban areas have been, to some extent, influenced by their food environment [15].

Nutrition epidemiological studies have extensively reported on the importance of diet quality and its association with the health of a population [16]. A priori plant-based diet (PBD) indices were developed by Satija et al., and they used a scoring system that reflected dietary intake relative to the distribution within a population [17,18]. These PBD indices graded food groups individually to distinguish between the nutritional value of plant foods that were healthy, e.g., whole grains (assigned positive scores), less healthy e.g., refined grains (assigned positive or reverse scores, depending on the index that was being calculated), and animal foods (assigned reverse scores). Moreover, several studies have shown that there were inverse and positive associations between healthy plant-based eating and cardiometabolic risk factors [19,20].

PBD indices, such as healthy dietary patterns, have been associated with reduced risks in various NCDs. Dietary patterns include both plant and animal foods, but the patterns emphasising the intake of healthy foods with low sugar, salt, and fat contents have generally been considered to be healthy. In contrast to a priori dietary methods, a posteriori exploratory methods, such as principal component analysis (PCA), have been applied in epidemiology to account for the variation in the diets of populations. This was a data-driven approach that was based on actual dietary intake and identified which dietary patterns were present in a studied population [21,22,23,24].

There is currently a gap in the literature regarding PBD adherence and dietary patterns in Africa and even more so in working-class males, such as commercial taxi drivers with unhealthy lifestyle habits. Therefore, the current study aimed to investigate the prevalence and the association between PBD indices and PCA-derived dietary patterns and their impact on the cardiometabolic risks of commercial taxi drivers. We, firstly, utilised the dietary data from an African population to determine the PBD adherence using three PBD indices (a priori analysis) and, secondly, assessed the association between these PBD indices and the cardiometabolic risk factors. Thirdly, we explored the associations between the PCA-derived dietary patterns (a posteriori analysis) and the cardiometabolic risk factors, including hypertension, dysglycaemia, dyslipidaemia, obesity, and subclinical inflammation.

## 2. Materials and Methods

### 2.1. Study Design and Population

This was a cross-sectional study using primary data collected in a South African cohort in the Western Cape province. The ongoing Street Foods Project aims to promote the adoption of healthier food items offered by street-food vendors in Cape Town, South Africa [25]. Commercial taxi drivers were recruited between 2019 and 2021 from two of the busiest transport routes in the Cape Metropole, including taxi drivers operating at taxi ranks in Bellville and Cape Town. The study participants were all males, 19 years or older, who consumed street food (≥3 days per week) sold by vendors in the Cape Metropole. Furthermore, taxi drivers who reported being diagnosed with or treated for any NCDs, such as hypertension and diabetes, were excluded from the study. These individuals were excluded because they were more likely to have altered their dietary habits than those who were undiagnosed or untreated. The Street Foods Project obtained ethical approval from the Biomedical Science Research Ethics Committee of the University of the Western Cape (Ref No: BM18/9/25). The current study was approved by the Health Research Ethics Committee of Stellenbosch University (S19/03/056).

### 2.2. Data Collection

A validated questionnaire was adapted from the first South African National Health and Nutrition Examination Survey 2012 (SANHANES-1) and administered to obtain data on the health status and dietary intake of commercial taxi drivers [26]. The study interviews were conducted face-to-face at the Health4Men Clinic consultation rooms located in Bellville and Cape Town. The following data were collected: sociodemographic factors, as well as anthropometric and blood pressure (BP) measurements.

Standardised techniques were used for the anthropometric measurements as recommended by the World Health Organisation (WHO). Weight measurements were taken using a calibrated scale that measured weight to the nearest 0.5 kilogram (kg). Height was measured to the nearest centimetre (cm) using a calibrated stadiometer that was placed on an even surface. Brachial blood pressure (BP) was measured three times, at least 5 min apart, in a seated position, using an automated monitor (Omron M2, Estates, IL, USA).

Fasting blood specimens were collected between 7 a.m. and 10 a.m. for various biochemical markers, including glucose, high-density lipoprotein cholesterol (HDL-C), and triglycerides (TG). Low-density lipoprotein cholesterol (LDL-C) was calculated using the Friedewald formula [27]. Blood specimens were analysed at an ISO15189:2012 accredited laboratory.

### 2.3. Dietary Assessment

A quantified 24 h recall (24-HR) questionnaire was administered by a trained fieldworker. The 24-HR collected information on the type of foods and beverages consumed, how it was prepared (i.e., what was added to the foods and beverages), and how much was consumed during the past 24 h. Furthermore, the 24-HR included food items sold by street vendors and was used to assess the dietary intake of 10% of the study population. Therefore, we anticipated little-to-no variation in the food items that were available in the food environment of taxi drivers and did not deem it necessary to administer multiple recalls to capture their typical diet. The questionnaire used for dietary data collection included plant- and animal-based food groups. The dietary intake of the commercial taxi drivers was analysed using the MRC Food Finder 3 [28] to determine the number of grams per food item, the number of macronutrients (protein, carbohydrates, and fats), and the other essential micronutrients consumed.

#### 2.3.1. A Priori Dietary Analysis

PBD adherence was assessed using three PBD indices that were developed by Satija et al. [17]. We modified the PBD indices by excluding the vegetable-oils food group because none of our study participants reported any intake of this food group during the recall period. Therefore, the current study calculated the overall PBD index (PDI), healthy PBD index (hPDI), and unhealthy PBD index (uPDI) using the 17 food groups listed in Table S1. To calculate these PBD indices, the food items were assigned to food groups that were categorised as healthy plant foods, less healthy plant foods, and animal foods. The latter was carried out to distinguish between the nutritional values of healthy plant foods (whole grains, fruit, vegetables, legumes, nuts, tea, and coffee), less healthy plant foods (refined grains, potatoes, fruit juices, sugar-sweetened beverages (SSBs), sweets, and desserts), and animal food groups (animal fat, egg, dairy, fish/seafood, meat, and miscellaneous animal foods). The grams consumed per food item were converted to servings sizes using the South African Food-Based Dietary Guidelines [29]. A relative scoring system was utilised to calculate the PDI, hPDI, and uPDI of each study participant. Food groups were ranked into quantiles of consumption based on serving sizes and assigned positive or reverse scores. Due to the granularity of the current study’s dietary data intake, our consumption scores in Table 1 ranged from 1 to 2 (low or high consumption, respectively) or from 1 to 3 (low, medium, or high consumption, respectively), instead of from 1 to 5, as per the original grading of Satija et al. Subsequently, the scores of the 17 food groups were summed for each of the study participants to obtain their total score for each PBD index (PDI, hPDI, and uPDI).

In the current study, we focussed on assessing the distribution of nutrients, such as vitamins B2 and B12, calcium, iron, and zinc (minerals), and macronutrients, such as polyunsaturated fatty acids (PUFAs) and, in particular, omega-3 fatty acids, as these are usually associated with PBDs [30,31].

#### 2.3.2. A Posteriori Dietary Analysis

PCA was performed on the dietary intake reported (grams consumed per day) for 11 plant and 6 animal food groups to identify dietary patterns [24]. We conducted the Kaiser–Meyer–Olkin (KMO) test to measure the sample adequacy and Bartlett’s test of sphericity to determine whether the data were suitable for factor analyses. A KMO test value greater than 0.5 and a Bartlett’s test of sphericity with significance level less than 0.05 warranted PCA. Furthermore, scree plots were inspected, and extracted components with eigenvalues ≥1 were retained. Factor reduction was carried out utilising the orthogonal varimax rotation method to improve interpretability by displaying the factors (i.e., food items/groups) that were correlated (i.e., load well together).

### 2.4. Definitions and Calculations

PBD adherence was assessed using three a priori dietary indices, namely the PDI, hPDI, and uPDI [17,32]. For the PDI, we assigned positive scores to all plant foods and reverse scores to animal foods. To calculate the hPDI, positive scores were assigned to the healthy plant foods and reverse scores to the less healthy plant and animal foods. However, for the uPDI, we assigned positive scores to the less healthy plants foods and reverse scores to healthy plant and animal foods.

PCA-derived dietary patterns were named using the food groups with factor-loading values ≥0.4 in the extracted components.

Common cardiometabolic risk factors were assessed, including hypertension, dysglycaemia, dyslipidaemia, obesity, and subclinical inflammation. Hypertension was defined as a systolic blood pressure (SBP) ≥ 140 mmHg and/or diastolic blood pressure (DBP) ≥ 90 mmHg [33,34]. Dysglycaemia was defined as fasting blood glucose (FBG) ≥ 7.0 mml/L [35,36]. Dyslipidaemia was defined by low HDL-C, raised LDL-C, and raised TG. HDL-C was defined as low according to a cut-off value of ≤1.0 mmol/L. Raised LDL-C was defined based on a cut-off value of ≥3.0 mmol/L. Raised TG was defined using a cut-off value of ≥1.5 mmol/L [37]. Obesity was defined using a BMI ≥ 30 kg/m^2^ [38]. Finally, hs-CRP levels between 3.0 and 10 mg/L were used to define subclinical inflammation [39,40,41].

### 2.5. Statistical Analysis

Descriptive analysis was performed to present the means with standard deviations for normally distributed variables; median and 25–75th percentiles for non-normally distributed variables; and counts and percentages for categorical variables. The Shapiro–Wilk normality test was utilised to assess the distribution of the data. Group comparisons used the Chi-squared test and equivalents for categorical variables; the Student’s *t*-test and analysis of the variance (ANOVA) for normally distributed variables; and non-parametric equivalents for skewed continuous variables. Multivariable logistic regressions were used to investigate the associations between PBD indices and derived dietary patterns, as well as the cardiometabolic risk profiles. We utilised categorical dietary variables (PBD indices were analysed as tertiles) with cardiometabolic risk profiles (categorical variables), while controlling for the effect of potential confounding variables, e.g., age and energy intake, amongst others. The odd ratios (ORs) and accompanying 95% confidence intervals (95% CIs) were reported. The IBM SPSS Statistics, version 27, software was used for data analysis, and *p*-values ≤ 0.05 were statistically significant.

## 3. Results

### 3.1. Characteristics of Study Participants

**Figure 1:**
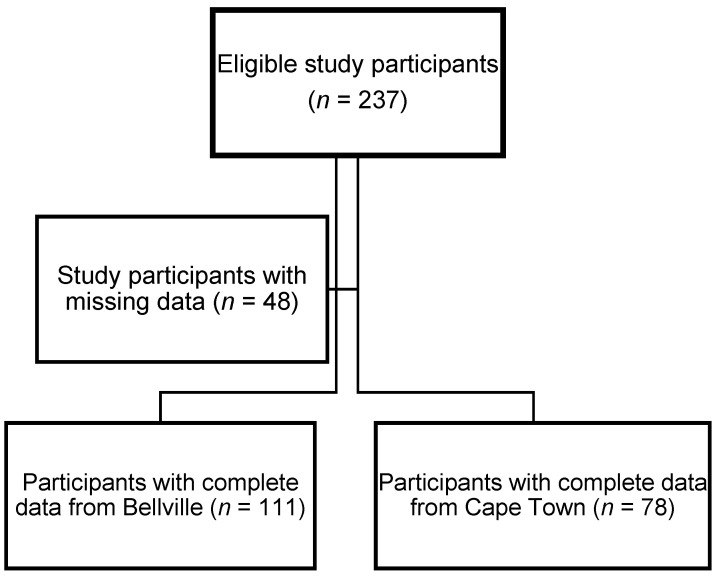
Flow diagram of participants included in the analytical sample.

The final sample consisted of 189 males with complete data. The study participants with missing variables for sociodemographic data or clinical measurements (*n* = 6); with no dietary data (*n* = 7); with no blood specimen or no blood test results due to insufficient sampling (*n* = 35) were excluded (Figure 1). Sixteen of the study participants had implausible energy intakes (<5000 kilojoules/day). However, we did not exclude these participants, as consideration was given to the sample size, and we subsequently adjusted for total energy intake in our analysis.

As displayed in Table 2, the study population had an overall median age of 38 years.

Overall, 52% of the taxi drivers were married or living as married, 44% were current smokers, and 54% were current drinkers. In terms of their cardiometabolic risk profiles, in descending order of prevalence, 40% of the taxi drivers had hypertension and low HDL-C; 37% had raised LDL-C and obesity; 30% had subclinical inflammation; 27% had raised TG; and 23% had dysglycaemia.

As shown in Table S1, the age variable was significantly higher in Cape Town, as compared to Bellville (*p* < 0.001). Taxi drivers in Bellville obtained a significantly higher level of education (*p* = 0.007), with 20% graduating from high school and 4% attending a tertiary institution, than those in Cape Town at 13% and 0%, respectively. The prevalence of hypertension was significantly different between the study areas (*p* = 0.020). Low HDL-C was also statistically significantly different between Bellville and Cape Town: 46% versus 31%, *p* = 0.036. None of the other cardiometabolic risk factors were significantly different between Bellville and Cape Town.

### 3.2. A Priori Analysis: PBD Indices

**Figure 2:**
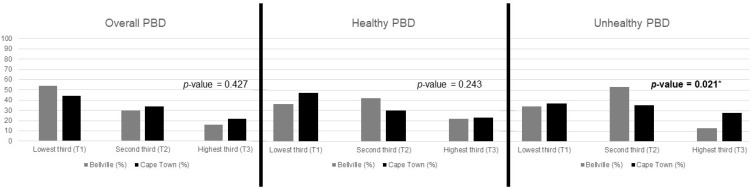
Prevalence of PBDs according to study area. Statistically significant *p*-values less than 0.05 are indicated in bold with an asterisk.

The distribution of the PBD indices by study area is shown in Figure 2, with significant differences between Cape Town and Bellville only in the uPDI (*p* = 0.021). The overall median scores for the PBD indices were a minimum score of 22 and a maximum score of 39 (see Table S2 in the Supplementary Materials).

Table 3 depicts the distribution of dietary factors that characterised the three PBD indices. Total carbohydrates (*p* = 0.003), sugars (*p* < 0.001), and fibre (*p* < 0.001) had statistically significant differences across the PDI. In addition, the intake of the following minerals was consistently greater in the top-third of the PDI: calcium (*p* < 0.001) and iron (*p* < 0.001). Although the distribution of zinc was significant (*p* = 0.038), it did not have a consistent pattern. In terms of the distribution of essential vitamins, there were statistically significant differences observed for vitamins B2 (*p* = 0.013) and D (*p* = 0.027).

In comparison, the hPDI showed a statistically significant distribution of nutrients, including all minerals and vitamins, as well as most macronutrients, except for fibre (*p* = 0.104).

There were fewer statistically significant distributed nutrients in the uPDI. Nonetheless, consistent patterns were observed in total protein (*p* = 0.046), carbohydrates (*p* < 0.001), monounsaturated fatty acids (MUFAs) (*p* = 0.003), and PUFAs (*p* < 0.001). However, inconsistent patterns were seen in total sugars (*p* < 0.001), fibre (*p* = 0.022), calcium (*p* = 0.035), and vitamin D (*p* = 0.010).

None of the other nutrients had a statistically significant distribution among the PBD indices.

### 3.3. Association between PBD Scores and Cardiometabolic Risk Profiles

Table 4 depicts the distribution of the cardiometabolic risk profile median scores of the PBD indices. There was a significant difference in the distribution of DBP in the uPDI (*p* = 0.026). However, there was no significant difference in the distribution of the PDI, hPDI, and uPDI for any of the other cardiometabolic measurements and biomarkers.

As presented in Table 5, there was a significant association between hPDI and raised TG. After adjusting for the education and marital status in model 2 and smoking and alcohol consumption in model 3, the association was slightly weaker, respectively, having a 1% lower likelihood of raised TG (*p* = 0.038), as compared to a 4% lower likelihood (*p* = 0.034) in the top-third of the results.

In the multivariable logistic regressions, there were no significant associations between the PDI and uPDI, and the cardiometabolic risk profiles.

### 3.4. A Posteriori Analysis: Dietary Patterns

**Figure 3:**
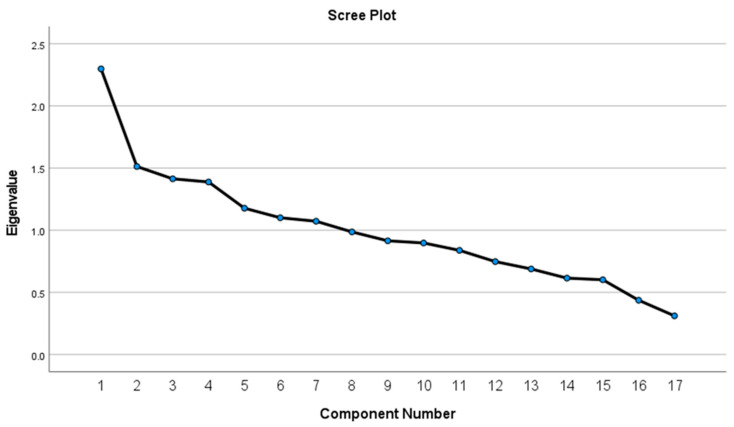
Scree plot displaying the eigenvalues of the food group components extracted by PCA.

The PCA extracted 7 components that explained 58.6% of the total variance in the diets of commercial taxi drivers who consumed street foods. The components with eigenvalues greater than 1.0, as depicted in the scree plot in Figure 3, were retained for further analysis.

Table 6 shows the factor-loadings of each of the retained components that were extracted to identify dietary patterns. Component 1 explained 11.3% of the total variance and had higher loadings for refined grains, meat, potatoes (positive loadings), and whole grains (negative loading). Component 2 explained 8.6% of the variance and was characterised by high loadings for fruit juices and fruit. However, the third component explained 8.2% of the variance and had higher loadings of legumes; sweets and desserts; nuts; SSBs; and animal fat. High loadings for fish/seafood, potatoes, and vegetables were retained in component 4 that explained 8.1% of the variance. Component 5 explained 7.6% of the variance and included higher loadings for the dairy and whole grains food groups. Component 6 explained 7.5% of the variance with higher loadings for the following food groups: tea and coffee; animal fat; sweets and desserts; and miscellaneous animal foods. SSBs, egg, vegetables, and nuts had high loadings in component 7, explaining 7.3% of the total variance.

Components 1–7 were labelled based on their factor-loadings with absolute values greater than 0.4: (1) refined grains and meat; (2) fruit juices, fruit and sweets, and desserts; (3) legumes and sweets and desserts; (4) fish/seafood, potatoes, and vegetables; (5) dairy and whole grains: (6) tea and coffee, and animal fat; and (7) SSBs and egg.

### 3.5. Association between PCA-Derived Dietary Patterns and Cardiometabolic Risk Profiles

Table 7 highlights the significant associations between specific identified dietary patterns and specific cardiometabolic risk factors. After adjusting for the sociodemographic factors, component 1 (i.e., the refined grains and meat pattern) was negatively associated with dysglycaemia with an AOR of 0.670, 95% CI: 0.447–0.998, and *p* = 0.049.

The fish/seafood, potatoes, and vegetables pattern (component 4), had a significant association with low HDL-C AOR of 1.41 and 95% CI: 1.03–1.92 in the model, which adjusted for age, study area, and energy intake (*p* = 0.030). Thereafter, when adjusting for sociodemographic factors, the association was maintained with an AOR of 1.43 and a 95% CI: 1.04–1.96. However, the association was attenuated after adjusting for the behavioural risk factors. This pattern was also positively associated with raised LDL-C values, which were only significant after adjusting for the sociodemographic and behavioural risk factors with an AOR of 1.44 and 95% CI: 1.04–1.99 (*p* = 0.027).

Component 7 presented a pattern that had high loadings of SSBs and egg, and it was positively associated with hypertension with an OR of 1.37 and, 95% CI: 1.00–1.86 (*p* = 0.047). However, this association was not sustained in the adjusted models. On the other hand, after adjusting for the sociodemographic and behavioural risk factors, there was a sustained association between the SSBs-and-egg pattern and subclinical inflammation: AOR = 1.53 and 95% CI: 1.08–2.16 (*p* = 0.017).

There were no significant associations between the other dietary patterns and the cardiometabolic risk profiles, such as component 2 (fruit juices, fruit, and sweets and desserts), component 3 (legumes and sweets and desserts), component 5 (dairy and whole grains), and component 6 (tea and coffee and animal fat).

## 4. Discussion

The focus of the current study was to assess the associations between PBDs and PCA-derived dietary patterns with cardiometabolic risk profiles in commercial taxi drivers consuming street foods sold in the Cape Metropole in South Africa. To our knowledge, this was the first study assessing PBD adherence in an African population using an a priori approach to calculate the PBD indices established by Satija et al. and, therefore, bridging the current gap in the literature. Taxi drivers in the current study had a high prevalence of hypertension and low HDL-C levels and were less likely to have dysglycaemia. Greater adherence to the uPDI was associated with taxi drivers having higher levels of DBP. Taxi drivers who demonstrated a greater adherence to the hPDI had better lipid profiles with a 64% lower likelihood of having raised TG. In addition, three of the seven identified dietary patterns were significantly associated with specific cardiometabolic risk factors. The refined grains and meat pattern had a negative association with dysglycaemia. Positive associations were found between the fish/seafood, potatoes, and vegetables pattern, and dyslipidaemia, i.e., low HDL-C and raised LDL-C. However, the SSBs-and-egg pattern had positive associations with hypertension and subclinical inflammation.

In the current study, we calculated three highly cited a priori PBD indices (PDI, hPDI, and uPDI) which have primarily been utilised by researchers in HICs to assess PBD adherence in large cohort studies, e.g., the NHANES study in the United States [17,18]. We were able to calculate these PBD indices in a South African adult population since most of the food groups included in the PDI, hPDI, and uPDI were common across geographical regions. More importantly, the dietary data collected in the current study fulfilled the criteria to construct these a priori indices [16,32]. The criteria being to, firstly, have dietary data on the intake of the relevant plant-and-animal food groups included in the PBD indices; secondly, having quantified dietary data to assess the consumption by quantiles; and lastly, applying the positive-and-reverse scoring system to calculate a total dietary score that could determine the adherence of an individual, relative to the population [18,43].

Our study also emphasised, yet again, the importance of adapting international indices that have been developed in HICs to reflect the traditional/cultural dietary habits of the studied population, e.g., a population from an upper-to-middle income country, such as South Africa, or other African and/or Asian LMICs. Dietary habits of LMICs differ substantially from the Westernised dietary habits of HICs [44,45]. For example, this was evident in the granularity of our dietary data, which concerned the food intake of street-food consumers (i.e., taxi drivers) who also had slightly different consumption patterns depending on the study population [14,15]. Food items that were commonly consumed in the current study belonged to the refined grains, SSBs, and meat food groups. These were frequently purchased as street foods, as they were readily available as energy-dense cooked meals, as compared to food items in the nuts; potatoes; fruit juices; sweets and desserts; animal fat; egg; dairy; fish/seafood; and miscellaneous animal foods, which were not purchased as frequently. Therefore, the dietary intake of our study cohort was skewed, and the quantiles of the consumption in the current study were lower than those of the original PBD indices [17,18]. Nonetheless, the consumption patterns in our cohort of commercial taxi drivers resembled that of male street-food consumers in our study setting [25].

In general, the prevalence of cardiometabolic risk profiles in the current study was comparable to other studies conducted in African taxi drivers [5,6]. Kurosaka et al. reported a 50.9% and 57.9% prevalence of diabetes mellitus and hypertension, respectively, in Japanese taxi drivers, which was greater than the prevalence in our cohort of South African taxi drivers [7]. The taxi drivers in our study had a lower prevalence of hypertension than those residing in other parts of South Africa and Ghana [5,6]. We did not find any literature on the prevalence of HDL-C in taxi drivers in other LMICs with which to compare the findings of the current study. The study from Japan reported similar lipid profiles to those of the taxi drivers in our study; since, the taxi drivers in Japan had low levels of HDL-C and high levels of LDL-C and TG [7].

Taxi drivers in the current study with a high adherence to the hPDI were less likely to have raised levels of TG. This finding was corroborated by reports that PBDs were beneficial and associated with lower TG levels, resulting in a reduced risk of NCDs such as dyslipidaemia and obesity [46,47]. These observed associations in our analysis could be attributed to hPDI highlighting the importance of healthy plant foods, such as whole grains, fruit, vegetables, nuts, and legumes. These foods are high in fibre, unsaturated fats, and other essential nutrients; hence, we speculated that they could confer protection against NCDs in individuals adhering to a healthy PBD [17,48].

On the other hand, our findings showed that greater uPDI adherence had a positive association with the levels of DBP in the taxi drivers. This was likely due to the high consumption of less healthy plant foods that received positive scores in the uPDI. According to the literature, these less healthy plant foods included refined grains, potatoes, fruit juices, SSBs, and sweets and desserts, and these energy-dense highly processed foods with high contents of saturated fat, salt, and sugar contributed to the rise in NCDs [17,23,48].

The uPDI focused on less healthy plant foods, which was characteristic of the PCA-derived dietary patterns that we identified in the current study. These dietary patterns were characterised by higher loadings of less healthy plant foods and animal foods. The dietary patterns consisted of plant foods that were nutrient-poor and energy-dense, such as refined grains (from the refined grains and meat pattern), potatoes consumed as French fries (from the fish/seafood, potatoes, and vegetables pattern), and cold/energy drinks (from the SSBs and egg pattern) [17,23]. These dietary patterns had statistically significant associations with specific cardiometabolic risk factors.

The dietary patterns identified had loadings of food items that included the consumption of mixed dishes. In the sections to follow, we provide a more detailed description of the food groupings before discussing the significant diet–disease associations among the derived dietary patterns in the current study. To elaborate on these dietary patterns, we utilised evidence from previous studies on street-food-consumption patterns in our setting [14,25,49].

Component 1 had the highest loadings of the refined-grains food group and included white bread, maize meal, and samp, amongst other starchy plant foods, that are usually consumed together with animal-protein food items, such as chicken, beef, etc. [14,25,49].

The refined grains and meat food groups in the current study consisted of refined grains (rice or mealie meal); fat cakes fried in oil with meat fillings; chicken or beef; and other meats/meat products consumed with skin and visible fat, which had been corroborated by existing literature on the street foods sold in the study area [49]. Our study observed that the refined grains and meat dietary pattern was associated with a 33% lower risk of dysglycaemia, which agreed with the findings of a study on meat consumption and dysglycaemia in Saudi Arabian males [50]. However, this finding was contrary to most evidence that foods, such as red and processed meats, increase the risk of cardiometabolic risk factors, such as dysglycaemia [51].

In our current analysis, we could not differentiate whether the type of meat (red or white) or the preparation (processed or unprocessed, with or without fat) was the mediating factor of this inverse association, because all these food items were categorised together in the meat food group of the PBD indices [17,18]. However, based on the recent literature, we speculated that the association could have been attributed to the consumption of unprocessed chicken consumed without the skin. Furthermore, the evidence from a review by Damigou et al. also discussed the possible influence of mixed dishes (food combinations), such as meat stews served with rice as a refined grain. Contrary to reports in the literature, we found that food combinations appeared to be beneficial rather than harmful [50]. This could be explained by dietary patterns that considered the type of meat (white versus red meat) and amount of fat, as compared to the proportions (serving sizes), where meat-to-vegetables (larger portions) reduced the cardiometabolic risks and refined grains (smaller portions) increased the cardiometabolic risks [52].

The two food groups that were highly correlated in component 4 (fish/seafood and potatoes) consisted of the following food items: fried fish and commercial French fries. These food items were commonly consumed as a meal readily purchased from fast-food outlets, including street-food vendors [14,25,49].

The dietary patterns with high loadings of fish/seafood, potatoes, and vegetables increased the likelihood of having low HDL-C and raised LDL-C. Based on our finding, mixed dishes (i.e., fish and chips) could be detrimental to the health of taxi drivers and, potentially, to other street-food consumers, even if fish and chips were consumed with a healthy salad consisting of various raw vegetables. There has been evidence that the fish/seafood, potatoes, and vegetables pattern reflected the consumption of common street-food items, such as fish and potato chips, with high fat and salt contents. These street-food items are prepared with oil to fry fish coated in batter, and potato chips are generally consumed with salty additives, such as spices and sauces [49].

Though the current study found a positive association between mixed dishes, such as fish and chips, some mixed dishes could promote healthy lipid profiles in taxi drivers. We believe that such health benefits could be attributed to the consumption of street-food items with high fibre and low fat content, such as whole-wheat brown bread and possibly a mixed breakfast meal, such as muesli (whole grains) with yoghurt (dairy). This phenomenon has been supported by studies reporting on the properties of healthy plant foods while not excluding animal foods from the overall diet [23]. Moreover, healthier street-food mixed dishes, such as yoghurt and muesli or samp and beans (grains and legumes), should, perhaps, be made available by street vendors and encouraged among their consumers [25,53].

Component 7 had high loadings in SSBs and egg. Based on our dietary intake data, the eggs were typically fried, rather than boiled or poached, and SSBs were often consumed as the preferred drink with any given meal for lunch, dinner, and/or even breakfast [14,25,49].

The SSBs-and-egg pattern had strong positive associations with hypertension and subclinical inflammation. This pattern had high loadings of street-food items, such as cold drinks and energy drinks with high sugar contents, and animal fats and proteins, such as eggs. Foods with high sugar and saturated fat contents are known to be positively associated with cardiometabolic risk factors, e.g., the indirect effect of fructose via elevated uric acid on the renin-angiotensin system to promote hypertension and inflammatory states, including obesity, which were relatively common in our study setting [54,55]. Our findings agreed with some of the literature from HIC and LMICs indicating that foods high in sugar, salt, and fat promoted NCDs [15].

In the current study, eggs were frequently fried in oil, rather than prepared with water as boiled or poached eggs. Therefore, the anti-inflammatory and other beneficial effects of egg consumption could have been negated in our study population [56]. Moreover, although eggs are a good source of protein, the literature has reported that the consumption of animal protein with pro-inflammatory properties was associated with an increased risk of NCDs [57,58]. On the other hand, based on a meta-analysis of randomised control trials, the consumption of eggs had no significant effect on inflammatory markers, including hs-CRP [56], which was used to define subclinical inflammation in the current study.

The current study did not find any statistically significant associations with the patterns of fruit and fruit juices, as well as legumes and sweets and desserts. Nonetheless, these dietary patterns, together with the SSBs-and-egg pattern, were somewhat comparable to the street-food patterns that had previously been described in our study setting. Hill et al. previously reported that frequently purchased street foods in Cape Town included fruit (87%); cooked food and baked products (72%); cold drinks (67%); and sweets (44%) [25]. This corroborated the observation that the PCA-derived dietary patterns from the current study were representative of the local food environment and promoted the unhealthy dietary habits of taxi drivers.

Although study area comparisons were beyond the scope of the current study and limited due to our relatively small sample, we noted some important statistically significant differences between the study areas, such as age, education, and the prevalence of hypertension and low HDL-C, as these may be worth exploring. These findings suggested that commercial taxi drivers operating in Bellville could have been exposed to healthier diets than those in Cape Town, e.g., via cooked plant-based street foods that were minimally processed and, thus, reduced the risk of NCDs. It further highlighted the crucial role of street vendors in the Cape Metropole, who had previously benefited from training and equipment to promote the consumption of healthy food in the food environment of commercial taxi drivers [53]. We, therefore, speculated that having a higher level of education and, possibly, a better understanding of nutritional and health information could help to prevent NCDs in commercial taxi drivers. This was supported by the literature on educational attainment as an important risk factor that influenced a population’s health status [8].

To our knowledge, this was the first study to characterise PBDs in a cohort of male adult commercial taxi drivers in South Africa within the context of other LMICs. One of the strengths of this study was its contribution to the literature on the associations between PBDs and NCDs, such as the cardiometabolic risk profiles in Africa. Additionally, the current study assessed the dietary habits of commercial taxi drivers using rigorous a priori and a posteriori dietary methods that had been selected based on evidence-based literature. These dietary methods allowed us to explore and characterise PBD adherence in commercial taxi drivers and identify dietary patterns that could elucidate the food groups that were more likely to be associated with a predisposition for nutrition-related NCDs. Utilising the 24-HR as a dietary assessment method was a strength in that it was piloted in the study sample to include all food items (i.e., street foods) that were consistently sold to taxi drivers in their food environment by street-food vendors.

We acknowledged that this study assessed dietary intake only on a single occasion using a 24-HR, which could have introduced recall bias and overlooked day-to-day variation. This could have affected the ranking of PBD adherence in some of our study participants. In addition, the granularity of the dietary intake data made it impossible to create quintiles of consumption, as per the original PBD indices developed by Satija et al. [17]. Nonetheless, we reported our scoring criteria to promote transparency and encourage the reproducibility in other study settings, such as LMICs that may have similar dietary habits. Another limitation of the current study was that we did not account for the levels of physical activity, which could have been a confounding variable that influenced the type of food and amount of food intake in our study participants. However, the current study was not focused on comparing the total energy intake versus output, which could be influenced by physical activity, but rather on the specific macro- and micro-nutrient intake of PBDs. The cross-sectional nature of this study was another limitation because we were unable to determine the cause–effect associations. Furthermore, our study findings are not generalisable to females and might lack some statistical power due to our small sample size.

## 5. Conclusions

PBDs exist in various forms that can be determined using a priori and a posteriori dietary methods. Our study showed that PBD adherence was a common practice among African populations. With minor modifications, we were able to calculate three PBD indices in a South African adult population that appeared to be following unhealthy PBDs rather than overall or healthy PBDs. Depending on the cooking methods, PBDs can be beneficial: healthy PBDs (i.e., hPDI) were inversely associated with raised TG, and dietary patterns containing refined grains and meats were inversely associated with dysglycaemia. On the other hand, PBDs can be harmful: unhealthy PBDs (i.e., uPDI) were positively associated with higher levels of DBP, and dietary patterns with high loadings of fish/seafood, potatoes, and vegetables were positively associated dyslipidaemia. SSBs-and-egg consumption appeared to be positively associated with hypertension and subclinical inflammation. However, these findings could have been influenced by the dietary habits specific to our study population as male street-food consumers that worked in the taxi industry. Therefore, we recommend that future studies further explore the prevalence and the associations between PBDs and dietary patterns with cardiometabolic risk profiles in other populations, i.e., male and female adults with sedentary lifestyles in other provinces of South Africa, as well as other African LMICs.

## Figures and Tables

**Table 1:** Scoring of the food groups used to calculate the PBD indices.

Food Groups	Number of Servings	Quantiles of Consumption	Scoring System
Whole grains	0	Low consumption = 1	HEALTHY PLANT FOODS PDI: positive scores hPDI: positive scores uPDI: reverse scores
	0.01–4.00	Med consumption = 2	
	4.01–12.63	High consumption = 3	
Fruits	0	Low consumption = 1	
	0.01–1.00	Med consumption = 2	
	1.01–8.42	High consumption = 3	
Vegetables	0	Low consumption = 1	
	0.01–1.00	Med consumption = 2	
	1.01–5.26	High consumption = 3	
Nuts	0	Low consumption = 1	
	0.01–3.16	High consumption = 2	
Legumes	0	Low consumption = 1	
	0.01–0.55	Med consumption = 2	
	0.56–2.11	High consumption = 3	
Tea and coffee	0	Low consumption = 1	
	0.01–1.00	Med consumption = 2	
	1.01–4.21	High consumption = 3	
Refined grains	0.00–5.00	Low consumption = 1	LESS HEALTHY PLANT FOODS PDI: positive scores hPDI: reverse scores uPDI: positive scores
	5.01–8.00	Med consumption = 2	
	8.01–37.89	High consumption = 3	
Potatoes	0	Low consumption = 1	
	0.01–1.00	Med consumption = 2	
	1.01–5.26	High consumption = 3	
Fruit juices	0	Low consumption = 1	
	0.01–2.11	High consumption = 2	
SSBs	0	Low consumption = 1	
	0.01–1.00	Med consumption = 2	
	1.01–6.32	High consumption = 3	
Sweets and desserts	0	Low consumption = 1	
	0.01–8.42	High consumption = 2	
Animal fat	0	Low consumption = 2	ANIMAL FOODS PDI: reverse scores hPDI: reverse scores uPDI: reverse scores
	0.01–6.32	High consumption = 1	
Egg	0	Low consumption = 2	
	0.01–6.32	High consumption = 1	
Dairy	0	Low consumption = 3	
	0.01–1.00	Med consumption = 2	
	1.01–5.26	High consumption = 1	
Fish/seafood	0	Low consumption = 2	
	0.01–4.21	High consumption = 1	
Meat	0.00–2.00	Low consumption = 3	
	2.01–4.00	Med consumption = 2	
	4.01–14.74	High consumption = 1	
Miscellaneous animal foods	0	High consumption = 1	
	0.01–4.21	Low consumption = 2	

PDI: Overall plant-based diet index. hPDI: healthy plant-based diet index. uPDI: unhealthy plant-based diet index.

**Table 2:** Characteristics of the commercial taxi drivers.

Parameters	Overall
Sociodemographic risk factors	
Age in years, median (25th percentile; 75th percentile)	38 (32–49)
Education, *n* (%)	
No schooling	7 (4)
Attended primary school	55 (29)
Attended high school	91 (48)
Matriculated (Grade 12)	32 (17)
Diploma	4 (2)
Marital status, *n* (%)	
Single/separated/divorced	90 (48)
Married/living as married	99 (52)
Behavioural risk factors	
Current smoker, *n* (%)	83 (44)
Current alcohol drinker, *n* (%)	102 (54)
Cardiometabolic risk factors	
Hypertension, *n* (%)	74 (40)
Dysglycaemia, *n* (%)	43 (23)
Low HDL-C, *n* (%)	75 (40)
Raised LDL-C, *n* (%)	70 (37)
Raised TG, *n* (%)	51 (27)
Obesity, *n* (%)	69 (37)
Subclinical inflammation, *n* (%)	57 (30)

Data presented as counts and percentages or medians (25th; 75th percentile). Hypertension: SBP ≥ 140 mmHg and/or DBP ≥ 90 mmHg, Dysglycaemia: FBG ≥ 7.0 mmol/L, Low HDL-C: levels ≤ 1.0 mmol/L, Raised LDL-C: levels ≥ 3.0 mmol/L, Raised TG: levels ≥ 1.5 mmol/L, Obesity: BMI ≥ 30 kg/m^2^, subclinical inflammation: hs-CRP between 3.0 and 10.0 mg/L.

**Table 3:** Distribution of dietary characteristics of the PBD indices.

Parameters	DRIs	Tertile 1	Tertile 2	Tertile 3	*p*-Value
PDI	RDA/AI *				
Macronutrients					
Total protein, g	56	106.6 (74.5; 138.7)	96.4 (71.9; 132.7)	127.0 (76.1; 173.9)	0.300
Plant protein, g		27.8 (18.4; 41.0)	30.5 (18.8; 48.0)	39.5 (21.0; 60.0)	0.126
Animal protein, g		56.4 (31.6; 85.7)	44.0 (26.3; 70.3)	44.0 (14.9; 65.9)	0.174
Carbohydrates, total, g	130	380.9 (250.1; 497.4)	362.2 (257.9; 575.0)	578.0 (366.7; 787.9)	**0.003**
Total sugars, g		23.0 (9.1; 48.6)	45.2 (20.3; 73.2)	69.3 (35.6; 107.9)	**<0.001**
Saturated fatty acids, g		20.06 (14.31; 30.20)	19.86 (12.06; 29.71)	21.52 (9.14; 47.38)	0.693
MUFA, g		22.99 (12.98; 43.68)	24.51 (14.91; 38.81)	38.10 (15.72; 54.82)	0.283
PUFA, g		21.44 (7.60; 37.55)	21.87 (10.60; 43.26)	21.35 (9.14; 47.38)	0.266
Total dietary fibre, g	30 */38 *	30.2 (20.7; 39.6)	32.4 (23.7; 55.7)	57.2 (45.4; 74.0)	**<0.001**
Minerals					
Calcium, mg	1000	604 (291; 839)	678 (355; 1120)	957 (701; 1206)	**<0.001**
Iron, mg	8.0	12.9 (9.8; 19.2)	16.1 (11.1; 29.7)	26.4 (17.1; 38.5)	**<0.001**
Zinc, mg	11.0	12.34 (8.87; 17.78)	12.18 (8.48; 18.73)	16.24 (10.60; 25.56)	**0.038**
Vitamins					
Vitamin B2, mg	1.3	1.37 (0.91; 1.95)	1.33 (1.01; 2.28)	1.88 (1.33; 2.88)	**0.013**
Vitamin B12, mcg	2.4 µg	3.6 (1.8; 7.1)	3.6 (1.6; 6.7)	3.2 (1.9; 6.8)	0.978
Vitamin D, mcg	5–15 * µg	6.48 (1.21; 13.79)	8.17 (1.73; 14.52)	10.75 (6.88; 15.49)	0.027
hPDI					
Macronutrients					
Total protein, g	56	139.5 (105.2; 191.0)	93.8 (76.3; 127.4)	71.8 (35.5; 96.3)	**<0.001**
Plant protein, g		36.2 (24.5; 45.9)	29.9 (16.3; 45.5)	20.6 (8.5; 33.1)	**<0.001**
Animal protein, g		76.9 (48.8; 101.0)	43.3 (28.1; 61.6)	21.0 (12.0; 45.3)	**<0.001**
Carbohydrates, total, g	130	525.1 (380.2; 753.8)	356.8 (253.1; 495.9)	279.6 (185.3; 378.2)	**<0.001**
Total sugars, g		48.6 (24.0; 92.4)	33.7 (12.2; 58.0)	24.9 (13.4; 59.4)	**0.017**
Saturated fatty acids, g		28.44 (21.26; 44.11)	17.68 (12.36; 27.47)	10.95 (6.57; 18.03)	**<0.001**
MUFA, g		43.68 (27.02; 62.94)	21.40 (13.30; 34.33)	13.05 (6.26; 19.94)	**<0.001**
PUFA, g		39.53 (24.88; 58.07)	18.22 (9.45; 39.75)	9.04 (4.57; 15.94)	**<0.001**
Total dietary fibre, g	30 */38 *	40.2 (26.7; 51.5)	32.5 (20.9; 45.4)	29.9 (22.3; 50.2)	0.104
Minerals					
Calcium, mg	1000	813 (535; 1130)	619 (354; 965)	585 (232; 949)	**0.020**
Iron, mg	8.0	18.6 (12.9; 31.0)	14.6 (10.7; 23.9)	13.0 (9.8; 19.1)	**0.001**
Zinc, mg	11.0	16.93 (12.34; 23.10)	12.01 (8.69; 18.14)	9.84 (5.98; 12.13)	**<0.001**
Vitamins					
Vitamin B2, mg	1.3	1.76 (1.29; 2.72)	1.34 (0.86; 2.04)	1.17 (0.71; 1.58)	**<0.001**
Vitamin B12, mcg	2.4 µg	5.4 (2.9; 15.4)	3.6 (1.6; 5.8)	2.2 (1.2; 3.4)	**<0.001**
Vitamin D, mcg	5–15 * µg	10.44 (4.86; 18.12)	6.59 (1.21; 13.70)	7.12 (1.22; 12.76)	**0.010**
uPDI					
Macronutrients					
Total protein, g	56	98.2 (75.3; 130.1)	104.4 (56.5; 142.7)	122.4 (85.0; 202.9)	**0.046**
Plant protein, g		29.0 (19.2; 42.4)	30.6 (16.0; 45.3)	32.2 (23.2; 42.8)	0.776
Animal protein, g		46.8 (26.3; 65.7)	49.0 (27.5; 76.9)	56.4 (30.3; 126.0)	0.116
Carbohydrates, total, g	130	365.5 (273.9; 481.3)	370.2 (238.3; 528.9)	597.4 (379.6; 756.8)	**<0.001**
Total sugars, g		31.0 (16.7; 64.6)	25.5 (9.4; 52.3)	68.8 (46.6; 169.6)	**<0.001**
Saturated fatty acids, g		18.03 (13.50; 25.54)	20.59 (9.58; 30.44)	24.63 (15.51; 44.84)	0.054
MUFA, g		20.80 (13.96; 38.75)	23.79 (11.71; 38.81)	47.43 (18.31; 70.07)	**0.003**
PUFA, g		15.83 (9.04; 28.44)	21.79 (8.15; 41.00)	44.08 (18.13; 76.12)	**<0.001**
Total dietary fibre, g	30 */38 *	37.5 (28.3; 50.9)	29.1 (18.4; 44.7)	42.0 (25.6; 54.2)	**0.022**
Minerals					
Calcium, mg	1000	782 (515; 1061)	545 (266; 965)	803 (509; 1040)	**0.035**
Iron, mg	8.0	15.1 (12.3; 24.3)	15.3 (9.6; 23.1)	18.6 (11.4; 35.5)	0.118
Zinc, mg	11.0	12.01 (10.11; 17.37)	13.00 (7.55; 19.00)	16.42 (9.27; 27.24)	0.088
Vitamins					
Vitamin B2, mg	1.3	1.49 (1.17; 1.92)	1.34 (0.76; 2.01)	1.45 (0.99; 3.09)	0.429
Vitamin B12, mcg	2.4 µg	3.5 (2.3; 5.1)	3.5 (1.2; 6.9)	3.5 (1.7; 16.8)	0.577
Vitamin D, mcg	5–15 * µg	9.50 (6.55; 15.50)	5.50 (0.89; 13.70)	7.74 (2.36; 14.49)	**0.010**

Data presented as medians (25th; 75th percentile). *p*-values with bold text are statistically significant, level of significance set at less than 0.05. PDI: overall plant-based diet index. hPDI: healthy plant-based diet index. uPDI: unhealthy plant-based diet index. MUFA: monounsaturated fatty acid. PUFA: polyunsaturated fatty acid. DRIs: Dietary reference intakes [42]. RDA: Recommended dietary allowance. AI: Adequate intakes. The adequate intakes are indicated with an asterisk *.

**Table 4:** Cardiometabolic risk profiles of the PBD indices.

Parameters	Normal Range	Tertile 1	Tertile 2	Tertile 3	*p*-Value
PDI					
SBP, mmHg	≤140	134 (122; 143)	132 (126; 139)	125.50 (116.00; 145.00)	0.470
DBP, mmHg	≤90	84 (74; 92)	80 (76; 91)	81.50 (73.00; 97.00)	0.905
FBG, mmol/L	≤7.0	5.77 (5.15; 6.67)	5.83 (5.06; 7.36)	5.47 (5.11; 6.68)	0.782
HDL-C, mmol/L	≥1.0	1.05 (0.93; 1.12)	1.07 (0.89; 1.34)	1.04 (0.87; 1.25)	0.671
LDL-C, mmol/L	≤3.0	2.76 (2.23; 3.29)	2.59 (2.17; 3.29)	2.78 (2.44; 3.24)	0.481
TG, mmol/L	≤1.5	1.06 (0.72; 1.46)	1.03 (0.62; 1.51)	1.10 (0.81; 1.68)	0.492
BMI, kg/m^2^	≤30	28.05 (23.44; 32.13)	28.25 (24.90; 32.74)	27.91 (24.11; 32.88)	0.961
hs-CRP, mg/L	≤3.0	2.60 (1.10–5.10)	2.20 (1.10; 4.90)	2.20 (1.20; 4.90)	0.969
hPDI					
SBP, mmHg	≤140	133.00 (123.00; 145.00)	132.00 (122.00; 140.00)	131.00 (121.00; 142.00)	0.636
DBP, mmHg	≤90	84.00 (79.00; 97.00)	79.50 (74.00; 90.00)	77.00 (71.00; 90.00)	0.056
FBG, mmol/L	≤7.0	5.50 (5.08; 6.52)	5.77 (5.06; 6.61)	6.22 (5.25; 7.36)	0.235
HDL-C, mmol/L	≥1.0	1.05 (0.95; 1.26)	0.99 (0.89; 1.20)	1.09 (0.99; 1.24)	0.352
LDL-C, mmol/L	≤3.0	2.69 (2.23; 3.28)	2.80 (2.28; 3.36)	2.55 (2.04; 3.20)	0.700
TG, mmol/L	≤1.5	1.04 (0.74; 1.47)	1.16 (0.64; 1.65)	0.99 (0.69; 1.35)	0.521
BMI, kg/m^2^	≤30	28.90 (25.42; 32.46)	27.96 (24.18; 31.05)	25.71 (21.71; 32.88)	0.172
hs-CRP, mg/L	≤3.0	2.70 (1.20; 5.90)	2.60 (1.10; 4.50)	2.20 (1.10; 4.80)	0.608
uPDI					
SBP, mmHg	≤140	134.00 (121.00; 143.00)	130.00 (121.00; 139.00)	138.00 (129.00; 145.00)	0.188
DBP, mmHg	≤90	80.00 (71.50; 91.00)	82.00 (75.00; 90.00)	90.00 (80.00; 100.00)	**0.026**
FBG, mmol/L	≤7.0	5.98 (5.13; 7.03)	5.72 (5.14; 6.85)	5.63 (4.99; 6.63)	0.966
HDL-C, mmol/L	≥1.0	1.05 (0.89; 1.27)	1.02 (0.89; 1.20)	1.09 (0.95; 1.39)	0.396
LDL-C, mmol/L	≤3.0	2.65 (2.28; 3.24)	2.87 (1.97; 3.29)	2.61 (2.23; 3.21)	0.783
TG, mmol/L	≤1.5	0.92 (0.70; 1.33)	1.04 (0.69; 1.61)	1.36 (0.92; 1.75)	0.095
BMI, kg/m^2^	≤30	26.54 (23.32; 32.63)	28.13 (23.60; 31.46)	30.66 (26.18; 32.64)	0.165
hs-CRP, mg/L	≤3.0	2.20 (1.10; 4.90)	2.30 (0.90; 4.80)	3.90 (1.50; 6.80)	0.136

Kruskal–Wallis test. Data presented as medians (25th; 75th percentile). *p*-values with bold text are statistically significant, level of significance set at less than 0.05. PDI: overall plant-based diet index. hPDI: healthy plant-based diet index. uPDI: unhealthy plant-based diet index. SBP: systolic blood pressure. DBP: diastolic blood pressure. FBG: fasting blood glucose. HDL-C: high-density lipoprotein cholesterol. LDL-C: low-density lipoprotein cholesterol. TG: triglycerides. BMI: body mass index. hs-CRP: high-sensitivity C-reactive protein.

**Table 5:** Associations between hPDI and cardiometabolic risk profiles.

**hPDI**	**Tertile 2**	**Tertile 3**	***p*** **-Value**
Hypertension	1.31 (0.65–2.68)	1.48 (0.65–3.46)	0.593
Model 1	1.14 (0.54–2.40)	1.42 (0.61–3.38)	0.718
Model 2	1.08 (0.50–2.31)	1.40 (0.59–3.40)	0.740
Model 3	1.01 (0.46–2.21)	1.37 (0.57–3.38)	0.755
Dysglycaemia	0.97 (0.40–2.34)	0.46 (0.18–1.12)	0.178
Model 1	0.88 (0.35–2.22)	0.41 (0.16–1.04)	0.144
Model 2	0.83 (0.33–2.11)	0.40 (0.15–1.03)	0.147
Model 3	0.85 (0.33–2.19)	0.41 (0.16–1.06)	0.159
Low HDL-C	0.64 (0.32–1.28)	1.62 (0.70–3.90)	0.090
Model 1	0.66 (0.32–1.37)	1.66 (0.71–4.06)	0.110
Model 2	0.62 (0.30–1.30)	1.57 (0.65–3.91)	0.115
Model 3	0.65 (0.30–1.37)	1.63 (0.68–4.11)	0.120
Raised LDL-C	0.95 (0.47–1.92)	1.43 (0.62–3.37)	0.611
Model 1	0.95 (0.46–1.98)	1.42 (0.62–3.39)	0.623
Model 2	0.98 (0.47–2.05)	1.43 (0.62–3.42)	0.646
Model 3	0.94 (0.45–1.97)	1.39 (0.60–3.35)	0.650
Raised TG	0.50 (0.23–1.08)	1.06 (0.41–2.90)	0.136
Model 1	0.40 (0.17–0.90)	0.97 (0.37–2.70)	0.053
Model 2	0.38 (0.16–0.86)	0.99 (0.37–2.81)	**0.038**
Model 3	0.36 (0.15–0.83)	0.96 (0.35–2.71)	**0.034**
Obesity	2.00 (0.97–4.20)	1.82 (0.80–4.28)	0.129
Model 1	1.74 (0.82–3.73)	1.72 (0.74–4.11)	0.269
Model 2	1.67 (0.78–3.62)	1.70 (0.73–4.11)	0.312
Model 3	1.62 (0.76–3.53)	1.66 (0.71–4.02)	0.358
Subclinical inflammation	1.95 (0.91–4.31)	1.12 (0.49–2.61)	0.206
Model 1	2.00 (0.90–4.59)	1.09 (0.47–2.61)	0.210
Model 2	1.96 (0.87–4.53)	1.12 (0.48–2.70)	0.242
Model 3	2.00 (0.88–4.70)	1.15 (0.48–2.79)	0.235

Data presented as unadjusted and adjusted odds ratios (ORs) with 95% confidence intervals. *p*-values with bold text are statistically significant, level of significance set at less than 0.05. Tertile 1 (the lowest third) was set as the reference category. Model 1—adjusted for age, study area, and energy intake. Model 2—adjusted for variables in Model 1 and sociodemographic risk factors such as marital status and education. Model 3—adjusted for variables in Models 1 and 2, and behavioural risk factors such as smoking and alcohol consumption. hPDI: healthy plant-based diet index. Hypertension: SBP ≥ 140 mmHg and/or DBP ≥ 90 mmHg. Dysglycaemia: FBG ≥ 7.0 mmol/L. Low HDL-C: levels ≤ 1.0 mmol/L. Raised LDL-C: levels ≥ 3.0 mmol/L. Raised TG: levels ≥ 1.5 mmol/L Obesity: BMI ≥ 30 kg/m^2^. Subclinical inflammation: hs-CRP between 3.0 and 10.0 mg/L.

**Table 6:** Seven components extracted and retained from the PCA.

**Food Groups**	**Component 1**	**Component 2**	**Component 3**	**Component 4**	**Component 5**	**Component 6**	**Component 7**
Whole grains	−0.416	−0.110	−0.066	−0.111	**0.702**	0.218	−0.012
Fruits	−0.131	**0.647**	−0.165	−0.002	0.179	−0.168	−0.030
Vegetables	−0.252	0.017	−0.022	**0.455**	−0.003	0.020	0.391
Nuts	0.053	−0.230	0.324	−0.228	−0.033	−0.050	−0.337
Legumes	0.110	−0.086	**0.663**	−0.126	0.004	−0.009	−0.085
Tea and coffee	−0.286	−0.005	0.108	0.185	0.160	**0.700**	−0.250
Refined grains	**0.845**	−0.038	−0.003	0.193	0.047	−0.092	−0.073
Potatoes	0.327	−0.172	0.246	**0.606**	0.135	0.108	−0.057
Fruit juices	−0.112	**0.755**	0.091	−0.040	−0.085	0.185	−0.085
SSBs	0.213	−0.202	0.304	−0.170	0.116	0.033	**0.649**
Sweets and desserts	0.055	**0.413**	**0.534**	0.110	0.028	0.320	0.174
Animal fat	0.288	−0.056	−0.509	−0.262	0.260	**0.408**	−0.022
Egg	−0.073	−0.018	−0.138	0.012	−0.122	−0.053	**0.642**
Dairy	0.211	0.197	0.002	0.082	**0.752**	−0.227	−0.056
Fish/seafood	0.049	0.071	−0.220	**0.708**	−0.072	−0.114	−0.063
Meat	**0.726**	−0.327	0.149	−0.124	−0.101	0.127	0.055
Miscellaneous animal foods	−0.133	−0.061	0.052	0.138	0.206	−0.550	−0.122

Factor-loadings with absolute values greater than 0.4 are indicated in bold text.

**Table 7:** Association between PCA-derived dietary patterns and cardiometabolic risk profiles.

Parameters	OR	95% CI	*p*-Value
Refined grains and meat pattern			
Hypertension	0.89	0.65–1.21	0.448
Model 1	0.86	0.62–1.18	0.340
Model 2	0.83	0.59–1.15	0.261
Model 3	0.782	0.56–1.10	0.156
Dysglycaemia	0.70	0.48–1.03	0.067
Model 1	0.69	0.47–1.02	0.062
Model 2	0.67	0.45–1.00	**0.049**
Model 3	0.67	0.44–1.00	0.051
Low HDL-C	0.95	0.70–1.28	0.713
Model 1	0.96	0.70–1.31	0.807
Model 2	0.94	0.68–1.28	0.685
Model 3	0.97	0.70–1.33	0.833
Raised LDL-C	0.97	0.71–1.31	0.829
Model 1	0.97	0.71–1.32	0.849
Model 2	0.97	0.71–1.32	0.845
Model 3	0.93	0.68–1.28	0.660
Raised TG	0.89	0.63–1.25	0.509
Model 1	0.87	0.62–1.24	0.450
Model 2	0.86	0.61–1.23	0.415
Model 3	0.83	0.58–1.19	0.314
Obesity	1.13	0.83–1.54	0.421
Model 1	1.12	0.82–1.54	0.476
Model 2	1.11	0.80–1.53	0.536
Model 3	1.07	0.77–1.49	0.674
Subclinical inflammation	1.00	0.73–1.39	0.979
Model 1	1.03	0.74–1.44	0.858
Model 2	1.03	0.73–1.45	0.866
Model 3	0.99	0.53–1.86	0.986
Fish/seafood, potatoes, and vegetables pattern			
Hypertension	1.17	0.86–1.58	0.320
Model 1	1.19	0.87–1.63	0.284
Model 2	1.17	0.84–1.61	0.349
Model 3	1.30	0.92–1.83	0.142
Dysglycaemia	0.91	0.63–1.31	0.606
Model 1	0.93	0.64–1.36	0.700
Model 2	0.91	0.62–1.33	0.630
Model 3	0.89	0.60–1.32	0.560
Low HDL-C	1.40	1.03–1.90	**0.034**
Model 1	1.41	1.03–1.92	**0.030**
Model 2	1.43	1.04–1.96	**0.026**
Model 3	1.37	0.99–1.90	0.055
Raised LDL-C	1.31	0.96–1.77	0.086
Model 1	1.31	0.97–1.78	0.081
Model 2	1.34	0.98–1.83	0.063
Model 3	1.44	1.04–1.99	**0.027**
Raised TG	0.88	0.62–1.24	0.457
Model 1	0.89	0.62–1.27	0.513
Model 2	0.87	0.60–1.25	0.455
Model 3	0.93	0.64–1.34	0.681
Obesity	1.08	0.80–1.47	0.613
Model 1	1.11	0.81–1.52	0.529
Model 2	1.09	0.79–1.51	0.585
Model 3	1.16	0.83–1.63	0.377
Subclinical inflammation	0.79	0.56–1.13	0.196
Model 1	0.81	0.56–1.17	0.268
Model 2	0.79	0.55–1.45	0.221
Model 3	0.76	0.52–1.13	0.176
SSBs and egg pattern			
Hypertension	1.37	1.00–1.86	**0.047**
Model 1	1.34	0.98–1.83	0.070
Model 2	1.34	0.97–1.85	0.074
Model 3	1.26	0.91–1.76	0.170
Dysglycaemia	1.19	0.86–1.66	0.302
Model 1	1.17	0.83–1.64	0.367
Model 2	1.17	0.84–1.64	0.361
Model 3	1.15	0.81–1.63	0.438
Low HDL-C	0.81	0.59–1.10	0.181
Model 1	0.82	0.60–1.11	0.199
Model 2	0.81	0.59–1.12	0.199
Model 3	0.87	0.63–1.20	0.393
Raised LDL-C	1.16	0.86–1.56	0.330
Model 1	1.16	0.86–1.57	0.334
Model 2	1.17	0.87–1.59	0.301
Model 3	1.19	0.87–1.63	0.283
Raised TG	1.09	0.79–1.50	0.615
Model 1	1.06	0.76–1.47	0.743
Model 2	1.05	0.76–1.46	0.766
Model 3	0.96	0.68–1.36	0.816
Obesity	1.07	0.79–1.44	0.671
Model 1	1.03	0.76–1.41	0.849
Model 2	1.03	0.75–1.40	0.868
Model 3	0.96	0.70–1.33	0.817
Subclinical inflammation	1.44	1.05–1.98	**0.023**
Model 1	1.46	1.05–2.04	**0.023**
Model 2	1.45	1.04–2.01	**0.029**
Model 3	1.53	1.08–2.16	**0.017**

Data presented as unadjusted and adjusted odds ratios (ORs) with 95% confidence intervals. *p*-values with bold text are statistically significant, level of significance set at less than 0.05. Model 1—adjusted for age, study area, and energy intake. Model 2—adjusted for variables in Model 1 and sociodemographic risk factors, such as marital status and education. Model 3—adjusted for variables in Models 1 and 2, and behavioural risk factors such as smoking and alcohol consumption. Hypertension: SBP ≥ 140 mmHg and/or DBP ≥ 90 mmHg. Dysglycaemia: FBG ≥ 7.0 mmol/L. Low HDL-C: levels < 1.0 mmol/L. Raised LDL-C: levels ≥ 3.0 mmol/L. Raised TG: levels ≥ 1.5 mmol/L. Obesity: BMI ≥ 30 kg/m^2^. Subclinical inflammation: hs-CRP between 3.0 and 10.0 mg/L.

## Data Availability

Data supporting the reported results will not be made available publicly and can be requested from T.L. or M.D.S.

## Supplementary Materials

The following supporting information can be downloaded at: https://www.mdpi.com/article/10.3390/nu15071789/s1, Table S1: Classification of food items by food groups. Table S2: Characteristics of commercial taxi drivers by study area. Table S3: Prevalence of PBDs according to study area.
