# Supplementary material for: The Association between Plant-Based Diets and Dietary Patterns with Cardiometabolic Risk in a Sample of Commercial Taxi Drivers in South Africa
Source: Nutrients. 2023 Apr 6;15(7):1789. doi: 10.3390/nu15071789 (PMC10096944; doi:10.3390/nu15071789)
Supplement: Supplementary file 1 [file nutrients-15-01789-s001.zip › nutrients-2276975-supplementary.pdf]

Table S1. Classification of food items by food groups

Food groups	Food items	Scoring system
Whole grains	whole grain oat & multigrain cereal; wheat bran breakfast cereal; muesli cereal; whole-grain wheat breakfast cereal; oatmeal (cooked); brown bread; whole wheat rolls	HEALTHY PLANT FOODS<br>PDI: positive scores<br>hPDI: positive scores<br>uPDI: reverse scores
Fruits	apple; avocado; banana; grape; mango (achar) peach; pear; pineapple; raspberry; fruit salad, fresh, without sugar (melon, orange, banana)	
Vegetables	mushroom (boiled); squash, butternut, (boiled, with sugar); broccoli (boiled); onion rings, breaded (fried); tomato sauce; tomato (raw); tomato, (fried in animal fat); lettuce (raw); cabbage (boiled); spinach (small leaved), boiled (USA); salad: French (lettuce, tomato, cucumber, no dressing); salad: mixed fresh vegetables (carrot, tomato, lettuce, no dressing); salad: coleslaw, commercial (USA); salad: Greek (lettuce, tomato, cucumber, olive, feta, no dressing); (salad: sweetcorn (mayonnaise, tomato, onion); (salad: sambal (tomato, onion); mixed vegetables, frozen, boiled (carrot, corn, peas, green beans, etc.); mixed vegetables, boiled, with polyunsaturated margarine (carrot, etc.); other vegetable (boiled); Soup, minestrone, commercial, prepared with water	
Nuts	Peanuts (roasted, salted); Peanut butter	
Legumes	Beans, dried, canned in tomato sauce (baked beans) (USA); Mixed dishes: Samp and beans; Biryani with sun oil (rice, lentils, spices, no potato, no meat)	
Tea and coffee	Tea, brewed (USA); Tea, herb, brewed (USA); Tea, rooibos, brewed; Coffee, brewed/instant (USA); Cream, fresh, coffee (12% fat) (USA); Malted milk beverage (FF, no sugar), e.g., Milo	
Refined grains	White bread; white bread flour (fortified); white rolls (fortified); Maize meal, super, porridge, stiff (white, unfortified); Maize meal, special, porridge, crumbly (white, unfortified); Baby cereal, mabele soft porridge, 6 months, original flavour (with added nutrients), dry; Ace Instant Porridge, Original Flavour (with added nutrients), dry cereal; corn breakfast cereal, plain; Breakfast cereal - Frosties, sugar-coated cornflakes; Breakfast cereal - Weet-bix; Breakfast cereal - puffed wheat, plain (USA); Rice, white, cooked (USA); Rice, white, cooked, with sun oil; Macaroni/Spaghetti, cooked (USA); Maize, samp/rice, cooked (white); Mixed dish: Samp and beans; Muffin, plain (UK); Cookies, commercial, plain; Vetkoek, home-made	LESS HEALTHY PLANT FOODS

	(cake flour, water); Roti, made with sun oil; Snack, savoury, average, e.g. Niknaks, Fritos, Ghost Pops	
Potatoes	Potato, boiled without skin; Potato chips/French fries, fried in sunflower oil (USA); Potato chips/French fries, frozen, heated in oven (USA); Snack, savoury, Chipniks; Snack, savoury, potato crisps/chips	PDI: positive scores<br>hPDI: reverse scores<br>uPDI: positive scores
Fruit juices	apple juice; grapefruit juice (canned, sweetened); grapefruit juice; orange juice (canned, sweetened); orange juice; peach juice; peach and orange juice	
SSB	Sugar, brown (USA); Score energy drink; Cold drink, Lucozade (UK); Cold drink, low-cal/artificially sweetened/diet squash, diluted (USA); Cold drink, squash, diluted; Cold drink, carbonated, average (e.g. Cola, Cream Soda, Tonic) (USA)	
Sweets and desserts	Sweets, fruit gum; Sweets, chewing gum; Sweets, peanut brittle (USA); Sweets, chocolate coated bar (UK); Sweets, chocolate, dark/bittersweet/Albany; Sweets, fudge/toffee/caramel; Scone, plain (FF, sun oil); Cookies, commercial, plain; Scone, plain (WM, HM); Pudding, dumplings (HM)	
Animal fat	Medium-fat spread, polyunsaturated; Floro light, Margarine, 50% polyunsaturated; Floro, Low-fat spread, polyunsaturated; Floro extra light, Medium-fat spread, <40% polyunsaturated; Margarine, brick/hard; Butter	ANIMAL FOODS<br>PDI: reverse scores<br>hPDI: reverse scores<br>uPDI: reverse scores
Egg	Egg, scrambled (LFM, sun oil); Egg, fried in sun oil; Egg, chicken, whole, boiled/poached	
Dairy	Maas/Sour milk, full fat; Milk, full fat/whole, fresh; Cheese, processed, full fat (UK); Cheese, processed, cheddar, high fat; Cheese, cheddar, high fat; Cheese, cheddar, white, high fat; Yoghurt, fruit, fat free, artificially sweetened (UK); Yoghurt, drinking, low fat, flavoured, sweetened (UK); Milk shake, vanilla, purchased (USA); Dairy-fruit juice mix	
Fish or seafood	Pilchard in tomato sauce (UK); Tuna salad (USA); Fish, low fat, battered/crumbed, fried in sun oil; Fish, medium fat, battered/crumbed, fried in sun oil	
Meat	Beef, brisket / regular mince, cooked – moist; Beef, loin, cooked – dry; Beef, chuck, cooked – moist; Beef, thick flank, cooked – moist; Beef, rib, wing, cooked – dry; Beef, rump, cooked – dry; Beef, mince (lean), savoury (tomato, onion); Biltong, beef (cured, dried) (USA); Chicken, meat only, frozen, boiled; Chicken, meat only, frozen, roasted;	

	Chicken, skin, fresh, cooked – moist; Chicken, meat and skin, frozen, boiled; Chicken, white meat, fresh, cooked – moist; Chicken, white meat, frozen, cooked – dry; Chicken, white meat, frozen, cooked – moist; Chicken, dark meat, fresh, cooked – dry; Chicken, dark meat, frozen, cooked – dry; Chicken, dark meat, frozen, cooked – moist; Chicken, batter dipped, fried (e.g. Kentucky) (USA); Chicken, giblets, curried; Chicken, giblets, cooked (simmered) (USA); Bacon, cured, pan-fried/grilled (USA); Pork, loin, grilled (chop) (USA); Schnitzel, pork chop (crumbed); Spaghetti bolognaise (lean mince); Stomach, lamb, cooked; Liver, chicken, cooked (simmered) (USA); Luncheon meat, beef / pork (USA); Meatball (lean mince, with egg); Mutton, leg (meat and fat), roasted (USA); Mutton, rib, grilled/roasted (USA); Offal, cooked (tripe / brawn / brain / tongue); Oxtail, stewed (meat only, salt added) (UK); Patty, beef, frozen, grilled (USA); Patty, chicken, crumbed / breaded, fried (USA); Polony / Bologna, beef and pork (USA); Sausage, smoked, beef and pork (USA); Sausage, beef & pork / boerewors, grilled (USA); Sausage, beef, grilled (UK); Sausage, beef, dry (UK); Salami, beef / pork (also Russians) (USA); Sausage roll, commercial, baked; Vienna sausage, beef and pork, canned (USA); Mixed dishes: Beef, stew, with cabbage; Beef, stew, with vegetables; Chicken (with skin), stew, tomato and onion; Chicken (with skin), stew, with vegetables; Chicken (with skin), curry; Chicken (without skin), curry; Mutton, curry; Mutton, stew, with vegetables; Mutton stew, with green beans; Pie, steak and kidney, commercial, baked; Samoosa, with mutton filling (UK); Spread (sandwich), pork / beef (USA); Soup, soup mix (with beef and vegetables); Soup, chicken cream, commercial, prepared with water; Soup, vegetable and beef, commercial, prepared with water; Soup, pea, split (with pork and vegetables); Soup, bean, dried (with beef and vegetables); Soup, chicken noodle, commercial, prepared with water (USA)	
Miscellaneous animal foods	Gravy, brown, powder, prepared with water (USA); Gravy, meat (fat-free stock, vegetables, thickened); Gravy, meat (20% fat, stock, thickened); Salad dressing, mayonnaise; Salad dressing, salad cream; Soup, asparagus cream, commercial, prepared with water	

Table S2. Characteristics of the commercial taxi drivers by study area

Parameters	Bellville	Cape Town	*p*-value
**Sociodemographic risk factors**			
Age in years	35 (30;43)	43 (35;50)	**<0.001**
Education, n (%)			
No schooling	7 (6)	0 (0)	
Attended primary school	24 (22)	31 (40)	
Attended high school	37 (47)	54 (49)	**0.007**
Matriculated (Grade 12)	22 (20)	10 (13)	
Diploma	4 (4)	0 (0)	
Marital status, n (%)			
Single/separated/divorced	55 (50)	35 (45)	
Married/living as married	56 (50)	43 (55)	0.526
**Behavioural risk factors**			
Current smoker, n (%)	54 (49)	29 (38)	0.136
Current alcohol drinker, n (%)	66 (60)	36 (46)	0.071
**Cardiometabolic risk factors**			
Hypertension, n (%)	35 (33)	39 (50)	**0.020**
Dysglycaemia, n (%)	22 (20)	21 (27)	0.251
Low HDL-C, n (%)	51 (46)	24 (31)	**0.036**
Raised LDL-C, n (%)	42 (38)	28 (36)	0.786
Raised TG, n (%)	25 (23)	26 (33)	0.099
Obesity, n (%)	35 (32)	34 (44)	0.090
Subclinical inflammation, n (%)	30 (27)	27 (35)	0.263

Data presented as counts and percentages or median (25th percentile;75th percentile). p-values with bold text are statistically significant, level of significance set at less than 0.05. Hypertension: SBP ≥ 140 mmHg and/or DBP ≥ 90 mmHg, Dysglycaemia: FBG ≥ 7.0 mmol/L, Low HDL-C: levels ≤ 1.0 mmol/L, Raised LDL-C: levels ≥ 3.0 mmol/L, Raised TG: levels ≥ 1.5 mmol/L, Obesity: BMI ≥ 30 kg/m^2, subclinical inflammation: hs-CRP between 3.0 and 10.0 mg/L.

Table S3. Prevalence of PBDs according to study area

Parameters	Overall	Bellville	Cape Town	*p*-value
**PDI**				
Observed range	22-35	22-34	23-35	
Median (25th;75th percentile)		26 (25;28)	27 (25;28)	0.094
Tertile 1, n (%)		51 (54)	33 (44)	
Tertile 2, n (%)		28 (30)	25 (34)	0.427
Tertile 3, n (%)		15 (16)	16 (22)	
**hPDI**				
Observed range	23-38	24-38	23-36	
Median (25th;75th percentile)		30 (27;31)	29 (27;31)	0.535
Tertile 1, n (%)		34 (36)	35 (47)	
Tertile 2, n (%)		39 (42)	22 (30)	0.243
Tertile 3, n (%)		21 (22)	17 (23)	
**uPDI**				
Observed range	25-39	25-37	25-39	
Median (25th;75th percentile)		32 (31;34)	33 (31-35)	0.337
Tertile 1, n (%)		31 (34)	27 (37)	
Tertile 2, n (%)		49 (53)	25 (35)	**0.021**
Tertile 3, n (%)		12 (13)	20 (28)	

Data presented as counts and percentages or median [25th percentile;75th percentile]. *p*-values with bold text are statistically significant, level of significance set at less than 0.05. PDI: overall plant-based diet index. hPDI: healthy plant-based diet index. uPDI: unhealthy plant-based diet index.
